# Response to: Simpson’s Paradox is suppression, but Lord’s Paradox is neither: clarification of and correction to Tu, Gunnell, and Gilthorpe (2008) by Nickerson CA & Brown NJL (https://doi.org/10.1186/1742-7622-5-2)

**DOI:** 10.1186/s12982-020-00089-7

**Published:** 2020-03-11

**Authors:** Mark S. Gilthorpe, Yu-Kang Tu

**Affiliations:** 1 Leeds Institute for Data Analytics, University of Leeds, Leeds, LS2 9NL UK; 2 Faculty of Medicine and Health, University of Leeds, Leeds, LS2 9LU UK; 3 Alan Turing Institute, British Library, London, NW1 2DB UK; 4 Institute of Epidemiology and Preventive Medicine, College of Public Health, National Taiwan University, Taipei, Taiwan

## Abstract

We commend Nickerson and Brown on their insightful exposition of the mathematical algebra behind Simpson’s paradox, suppression and Lord’s paradox; we also acknowledge there can be differences in how Lord’s paradox is approached analytically, compared to Simpson’s paradox and suppression, though not in every example of Lord’s paradox. Furthermore, Simpson’s paradox, suppression and Lord’s paradox ask the same *contextual* questions, seeking to understand if statistical adjustment is valid and meaningful, identifying which analytical option is correct. In our exposition of this, we focus on the perspective of context, which must invoke causal thinking. From a causal thinking perspective, Simpson’s paradox, suppression and Lord’s paradox present very similar analytical challenges.

We congratulate Nickerson and Brown for their exposition of the mathematics behind Simpson’s paradox, suppression and Lord’s paradox [1]. They explain the extent of statistical adjustment obtainable in assessing the $X$–$Y$ focal relationship when augmenting the linear model $Y\sim X$ with $Z$ to $Y\sim X + Z$: depending on the relationships amongst $X$, $Y$ and $Z$, the $X$ coefficient increases, decreases, or changes sign (hence ‘reversal paradox’).

Illustrated as Simpson’s paradox, this phenomenon explores the association between *any* two binary variables ($X$ and $Y$) that might be reversed if conditioned on *any* third binary variable ($Z$). Illustrated as suppression, this phenomenon involves $X$, $Y$ and $Z$ as *any* continuous variables. In both instances, the univariable linear model $Y\sim X$ and the multivariable linear model $Y\sim X + Z$ are contrasted and ‘reversal paradox’ occurs if the analytical options evaluating the $X$–$Y$ focal relationship conflict or differ. In contrast, Lord’s paradox is often illustrated for the specific context based on Lord’s 1967 paper [2], where $Z$ and $Y$ are *baseline* and *follow*-*up* measures of student weights, respectively, at the start and end of first year in college. The $X$–$Y$ focal relationship is the effect of *sex* ($X$) on *weight change* ($Y - Z$). This common understanding of Lord’s paradox exhibits different analytical options to Simpson’s paradox or suppression; contrasts are made between the model ($Y - Z\sim X + Z$) and *t* test of weight change ($Y - Z$) by *sex* ($X$). This is not mathematically identical to Simpson’s paradox or suppression. However, Lord published a note in 1969 in which he clarified that change-score analysis is possible only where such a quantity is calculable; [3] he urged reflection on where $Y$ and $Z$ are not on the same scale and options revert to the multivariable ($Y\sim X + Z$) verses univariable ($Y\sim X$) model, as for Simpson’s paradox and suppression. Whether examining follow-up ($Y$) or change-score ($Y - Z$), Lord’s paradox asks the *same question* to that for Simpson’s paradox and suppression: *is statistical adjustment of*$Z$*valid and meaningful?*

We did not discuss all analytical options in our paper since our focus was whether statistical adjustment is appropriate from a *causal inference* perspective [4]. Confusion arises if results from different evaluations of the same focal relationship differ or contradict. But there is no ‘paradox’ if the ‘correct’ or ‘meaningful’ analysis can be guided by *context*. Context need not be linked to the mathematics of analytical options. For Simpson’s paradox, suppression *and* Lord’s paradox the identical question is: *which analytical option is ‘correct’ or ‘meaningful’?*

The answer is not obtainable from data or mathematics, but from *contextual theory*. For instance, we assume that $X$ precedes $Y$, else there is no meaningful inference to be had. If $Z$ precedes $X$, then $Z$ ‘confounds’ the $X$–$Y$ focal relationship and statistical adjustment is warranted [5]. In contrast, if $X$ precedes $Z$, then $Z$ ‘mediates’ the $X$–$Y$ focal relationship and statistical adjustment is *not* warranted, unless seeking the *direct* effect of $X$ on $Y$ [5]. The mathematics and inferential context are equivalent for Simpson’s paradox and suppression, with variables either entirely categorical or continuous, respectively. For Lord’s paradox, in his 1967 paper [2], sex (at birth) precedes baseline weight (at entry to college), and baseline weight ($Z$) mediates the focal relationship between *sex* and *weight change*. We either adjust or not for baseline weight ($Z$); the same dichotomy for Simpson’s paradox and suppression.

There are additional complications with Lord’s paradox. Change-scores ($Y - Z$) with observational data do not generally provide meaningful *causal inference*, and the preferred approach is to view change as the part of $Y$ not explained by $Z$ [6]. The $X$-$Y$ focal relationship is thus more reliably estimated for the effect of sex ($X$) on follow-up weight ($Y$). The question is again whether to adjust for baseline weight ($Z$). The $X$ coefficients in $Y - Z\sim X + Z$ and $Y\sim X + Z$ are mathematically equivalent [7], whereas they differ for $Y - Z\sim X$ and $Y\sim X$ [6]. Despite multiple analytical options, there is none that yields *total* effect of sex on weight change.

Thinking more generally, as encouraged in the 1969 note, different contexts warrant different analytical strategies. Suppose, for instance, students are assigned to (mixed-sex) halls and the research question examines how halls affect weight change during the college year. Baseline weight ($Z$) precedes hall assignment ($X$), and $Z$ is a confounder or competing exposure if not causally related to $X$ (e.g. hall assignment is random). The multivariable models $Y - Z\sim X + Z$ and $Y\sim X + Z$ both appropriately (and equivalently) [7] estimate *total* effect of halls on weight change.

Lord’s paradox may be especially challenging and there are several analytical options [2], but Lord stressed the same *interpretational* issues as for Simpson’s paradox and suppression [3]. Understanding *context* is essential. In our exposition of this issue, we focus on the analytical validity according to *context* [4]. Although Nickerson and Brown provide an excellent exposition of the mathematics of statistical adjustment, analytical validity is more than algebra; robust and meaningful inference needs causal thinking [5], which is complementary.

## Data Availability

Not applicable
